# Quality of Life of Lung Cancer Patients with Immune-Related Endocrinopathies During Immunotherapy: A Prospective Study Based on the EORTC QLQ-C30 and QLQ-LC13 Questionnaires in Romania

**DOI:** 10.3390/curroncol32060332

**Published:** 2025-06-05

**Authors:** Simona Coniac, Mariana Cristina Costache-Outas, Ionuţ-Lucian Antone-Iordache, Alexandra-Valentina Anghel, Maria-Alexandra Bobolocu, Andreea Zamfir, Horia-Dan Liscu, Andreea-Iuliana Ionescu, Corin Badiu

**Affiliations:** 1 Department of Medical Oncology, Hospice Hope Bucharest, 023642 Bucharest, Romania; simona.horlescu@drd.umfcd.ro; 2 Department of Endocrinology, “Carol Davila” University of Medicine and Pharmacy, 020021 Bucharest, Romania; corin.badiu@umfcd.ro; 3 Department of Endocrinology, Coltea Clinical Hospital, 030167 Bucharest, Romania; costache.mariana@gmail.com; 4 Department of Radiotherapy, Coltea Clinical Hospital, 030167 Bucharest, Romania; 5 Discipline of Oncological Radiotherapy and Medical Imaging, “Carol Davila” University of Medicine and Pharmacy, 020021 Bucharest, Romania; alexandra-valentina.anghel0125@rez.umfcd.ro (A.-V.A.); alexandra.c.bobolocu@stud.umfcd.ro (M.-A.B.); andreea.zamfir@stud.umfcd.ro (A.Z.); andreea-iuliana.ionescu@umfcd.ro (A.-I.I.); 6 Department of Medical Oncology, Colțea Clinical Hospital, 030167 Bucharest, Romania; 7 “C.I. Parhon” National Institute of Endocrinology, 011863 Bucharest, Romania

**Keywords:** quality of life questionnaire, lung cancer, immune checkpoint inhibitors, endocrine immune-related adverse events, Romania

## Abstract

(1) Background: Globally, lung cancer is the leading cause of cancer death, but immunotherapy has impressively improved the outcomes, generating longer progression-free survival and overall survival. Endocrine immune-related adverse events (irAEs) are mostly irreversible and need life-long hormonal substitution therapy. The evaluation of the quality of life of lung cancer patients experiencing endocrine toxicities during immune checkpoint inhibitor (ICI) treatment is relevant for both patients and healthcare providers. (2) Methods: This was a prospective cohort study of lung cancer patients treated with immune checkpoint inhibitors in a tertiary-level hospital in Romania from 1 December 2021 to 30 September 2024. Quality of life was assessed using versions of the EORTC QLQ-C30 and EORTC QLQ-LC-13 validated and translated into the Romanian language. We investigated several clinical variables to evaluate their impact on QoL. (3) Results: Fifty-nine lung cancer patients were evaluated for their QoL before ICI initiation and at the end of the study. Endocrine-irAEs occurred in 17 lung cancer patients (28.8%). Quality of life as assessed using the EORTC questionnaires was statistically significantly improved, even in patients experiencing endocrine-irAEs. (4) Conclusions: Our prospective cohort study succeeded in delivering the proof of concept of an increased QoL in lung cancer patients who had developed endocrine-irAEs under immunotherapy. Despite toxicities, especially rather frequent endocrine-irAEs, ICIs enabled durable disease control and symptom relief, improving the QoL of the overall trial population. As more lung cancer patients undergo immunotherapy in metastatic or early stages, we draw attention to this particular patient population with autoimmune endocrinopathies, as they will live longer and require life-long hormonal therapy.

## 1. Introduction

Worldwide, lung cancer remained the most frequently diagnosed cancer and the leading cause of cancer death in males in 2022. Additionally, lung cancer was the second most commonly diagnosed cancer in women, rising from the third to the second leading cause of cancer, from 2020 to 2022 [1,2]. According to Globocan’s country–site-specific statistics, in Romania, lung cancer ranked second in its incidence in males and fourth in females but continued to be the foremost cause of cancer death in both sexes [3].

Immune checkpoint inhibitors (ICIs) have established a new cutting-edge therapy for lung cancer patients, with impressive efficacy in randomized clinical trials (RCTs). Humanized or fully human monoclonal antibodies specially designed to break cancer immune tolerance by restoring T cell recognition against tumor cells, ICIs dampen the cytotoxic T-lymphocyte-associated antigen 4 (CTLA-4) and the programmed cell death 1 (PD-1)/ligand (PD-L1) pathways. The clearly approved guideline recommendations of ICIs in unresectable locally advanced non-small-cell lung cancer (NSCLC) patients as consolidation therapy [4,5] or as the first- or second-line treatment in metastatic non-oncogene-addicted NSCLC patients [6,7,8,9,10,11,12,13] have positioned them as the mainstay of treatment. Even in more aggressive small-cell lung cancer (SCLC) patients, immunotherapy dramatically improved the overall survival in RCTs and consolidated the standard of treatment [14,15,16].

Nevertheless, the amplified use of ICIs in lung cancer patients has brought, besides benefits, a unique side effect profile of immune-related adverse events (irAEs) and completely different health-related quality of life concerns. Endocrine toxicities of immunotherapy have emerged as a distinctive group of irAEs having particular characteristics. Besides being more frequent than other irAEs, endocrine-irAEs rarely tend to be so severe as to stop immunotherapy but in fact become irreversible, and patients need life-long hormonal replacement therapy [17,18]. Furthermore, a significant positive impact of autoimmune endocrinopathies, especially thyroid disorders, on ICI efficacy has been evidenced, confirmed as improved progression-free survival (PFS) and overall survival (OS) in meta-analyses and real-life data on lung cancer patients [19,20,21]. The predictive power of endocrine-irAEs for the enhanced effectiveness of ICIs in lung cancer patients needs to be validated by more research on their quality of life because these patients live longer under both immunotherapy and endocrine autoimmune toxicities.

Medical professionals frequently under-detect symptoms or underestimate the side effects of oncological therapies, especially if not life-threatening, although these symptoms influence the quality of life of cancer patients [22,23]. In clinical practice, patient symptoms reported via questionnaires revealed a better quality of life (QoL) and toxicity management, an improved adherence to treatment, fewer visits to the emergency room or hospital admissions, and enhanced overall survival. However, the adoption of patient-reported outcome measures (PROMs) faces multiple challenges and barriers, such as the availability of trained healthcare providers, the accessibility and availability of resources, and patients’ levels of understanding and responsibility [24].

The European Organization for Research and Treatment of Cancer (EORTC) Core Quality of Life Questionnaire (QLQ-C30) and the EORTC Quality of Life Questionnaire-Lung Cancer 13 (QLQ-LC13) have been confirmed to have solid legitimacy in oncological clinical practice for more than twenty years in lung cancer patients [25]. Health-related quality of life research into ICIs still uses the QOL-C30 and QLQ-LC13 to evaluate, investigate and report lung cancer patients’ global health status and specific therapy or lung cancer symptoms [26]. Available on request for academic purposes, the QLQ-C30 and QLQ-LC13 have been translated into and validated in the Romanian language. To better understand and treat immune-related adverse events, the EORTC is currently developing a new quality of life questionnaire for cancer patients treated with immune checkpoint inhibitors, the QLQ-ICI, a useful tool for future investigation [27].

In our prospective observational exploratory study, we aimed to assess the quality of life of lung patients treated with immunotherapy in routine clinical practice in a tertiary-level hospital in Romania. We focused on multiple clinical variables that could impact QoL, especially endocrine immune-related adverse events.

## 2. Materials and Methods

### 2.1. The Study Population

This prospective observational non-interventional cohort study was conducted in a tertiary-level hospital, Colţea Clinical Hospital, Bucharest, Romania. We utilized the registered data of lung cancer patients treated with ICIs from 1 December 2021 to 30 September 2024.

The inclusion criteria for adult lung cancer patients involved the following:

- Non-oncogene-addicted advanced or metastatic non-small-cell lung cancer;
- Small-cell lung cancer;
- Signed informed consent to participate in this study;
- Agreement to immunotherapy according to the Romanian National Protocols.

The exclusion criteria comprised patients with the following characteristics at the baseline, before the start of ICI therapy:

- Renal failure;
- Liver failure;
- Untreated oncogenic driver mutations;
- Active Hepatitis B or Hepatitis C infection;
- Active human immunodeficiency virus (HIV) infection;
- Active autoimmune diseases or active immunosuppressive treatment;
- Pregnancy or lactation.

During this study, lung cancer patients were monitored under routine oncological practice and the frequencies of hospital visits respected specific ICI cycles as once every two weeks (q2w), once every three weeks (q3w) or as needed. The baseline demographic and clinical characteristics of the lung cancer patients, type of ICI, response to treatment and co-medication during ICI therapy were documented using registered data. The ICI efficacy was evaluated using imaging techniques following the Response Evaluation Criteria in Solid Tumors (RECIST) v1.1 [28].

All lung cancer patients were monitored using thyroid functional tests (TFTs) at baseline and before each ICI cycle, with at least one follow-up. Endocrine-irAEs were defined as any occurring autoimmune endocrinopathy during ICI therapy and were diagnosed, graded, treated and followed up by an endocrinologist in the MDT, following international guidelines [29,30]. For grading endocrine-irAEs, we referred to the Common Terminology Criteria for Adverse Events (CTCAE) v5.0 [31]. Immune-related thyroid disorder was defined as clinical or subclinical hypothyroidism or hyperthyroidism. Primary adrenal insufficiency (PAI) due to ICIs was described as a low morning cortisol level and high adrenocorticotropic hormone (ACTH) value or an abnormal cosyntropin (Synacthene) test. Immune-mediated hypophysitis was distinguished according to several hormonal axes, such as thyroid, adrenal or gonadotrophic deficiencies. Immune-related Diabetes Mellitus (DM) was characterized as worsening pre-existing DM or the new onset of insulin-dependent DM.

This study was approved by the Ethical Institutional Review Board of Coltea Clinical Hospital. This study was conducted in accordance with the Declaration of Helsinki. Informed consent was obtained from all of the patients who participated in this study and completed both questionnaires.

### 2.2. The Quality of Life Questionnaires and the Scoring Formula

Participating patients received and completed two quality of life surveys, one before ICI initiation and the second one in September 2024, at the end of the study period. Patients completed the questionnaires electronically in the following order: the QLQ-C30, then the QLQ-LC13. Versions of the quality of life questionnaires validated in and translated into the Romanian language were applied following receipt of the EORTC’s permission on 25th November 2021. The EORTC QLQ-C30 and EORTC QLQ-LC13 were applied to lung cancer patients to evaluate their quality of life during immunotherapy.

The QLQ-C30 consisted of thirty questions applicable to all cancer patients, aiming to evaluate their functional scale (physical, role, emotional, cognitive and social), symptom burden (fatigue, pain, nausea and vomiting, dyspnoea, insomnia, appetite loss, constipation, diarrhoea, financial issues) and global health status.

The QLQ-LC13 specifically measured lung-cancer-associated symptoms (cough, dyspnoea, hemoptysis and site-specific pain) and treatment-related symptoms (peripheral neuropathy, sore mouth, dysphagia and alopecia). Each question’s answer ranged from 1 (not at all) to 4 (very much). Each answer for the global health status ranged from 1 (very poor) to 7 (excellent). The range was defined by the difference between the highest and the lowest possible answers for each item, such as three for the functional and symptom scales and six for the global health status. Scoring manuals provided on the EORTC’s site had similar principles.

For all scales, the RawScore, RS, is the mean of the component items:

*RawScore* = *RS* = (*Item* 1 + *Item* 2 +...+ *Item n*)/*n*, *where n* = *item range*. (1)

Thus, for the functional scales

*Score* = [1 − (*RS* − 1)/*range*] × 100 (2)

and for the symptom scales/items and global health status/QOL,

*Score* = [(*RS* − 1)/*range*] × 100. (3)

A high score on the functional scale signified a high/good healthy level of functioning, so a higher score meant a better QoL. This was similar for the global health status/quality of life status. However, a high score for the symptom scale/a symptom item represented a high level of symptomatology/health issues, so a higher level of symptoms meant a worse QoL.

The main endpoint of this observational research was to assess the quality of life of lung cancer patients during immunotherapy. Additionally, the subsidiary outcome consisted of identifying the potential impact of endocrine immune-related adverse events upon the QoL of lung cancer patients.

### 2.3. Statistical Analysis

JASP 0.19.3 (JASP Team (2025), JASP (Version 0.19.3) [computer software]) was used for the statistical analysis. Categorical data are presented as numbers and percentages, while nominal data are presented as the mean and standard deviation.

In order to assess the differences between the QoL scores at the start of immunotherapy and at the study endpoint, Wilcoxon’s paired samples test was used. Mann–Whitney U tests and independent samples T-tests were used (depending on the outcome of the Shapiro–Wilk normality test) to investigate the differences in the QoL scores for several immunotherapy-related predictors.

Considering that we applied our questionnaires to patients who had been on ICIs for different periods of time, we built a multiple linear regression model in order to investigate whether the treatment duration significantly influenced QoL scores. We included the following predictors: baseline QoL scores, number of weeks on ICIs and age. The normality of the residuals was checked using Q-Q plots; homoscedasticity was checked using residuals vs. predicted plots; multicollinearity was assessed using the VIF; and the tolerance and correlations between residuals were checked using the Durbin–Watson test.

As our study was exploratory in nature and was intended to generate further hypotheses, we did not adjust for multiple comparisons in order to have a lower type II error. A *p*-value <.05 was considered statistically significant.

## 3. Results

### 3.1. The Study Population

**Figure 1:**
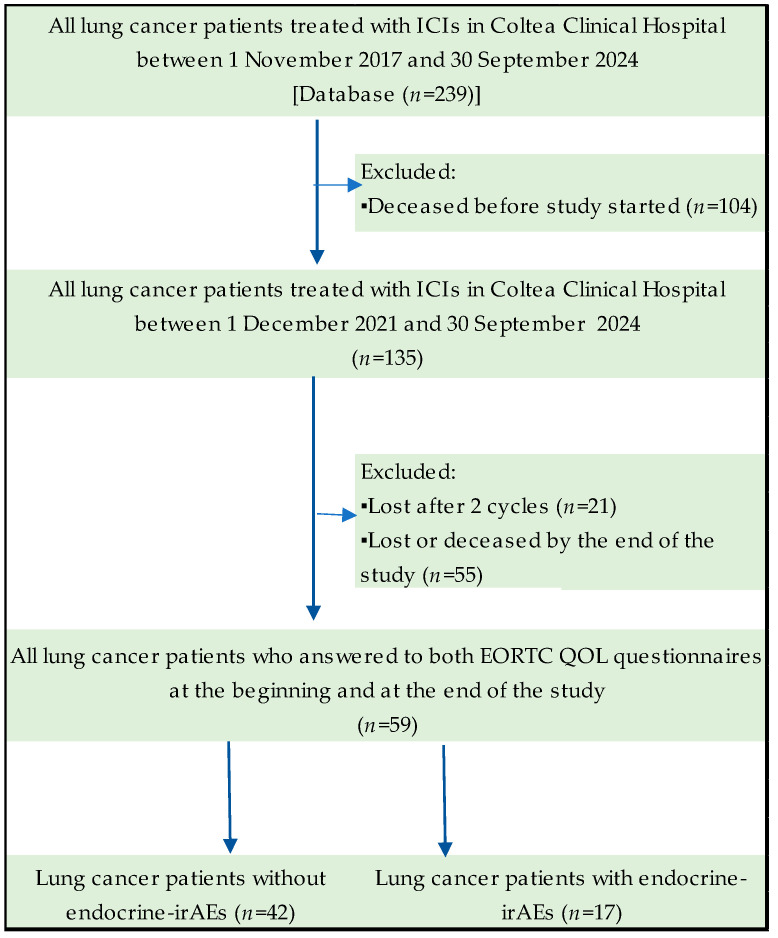
Flowchart of the study cohort.

Our study investigated a population of 59 lung cancer patients treated with immunotherapy in a tertiary-level hospital in Romania, Coltea Clinical Hospital. Of the entire cohort of patients treated in this clinic from 1 November 2017 to 30 September 2024, 135 were alive on 1st December 2021, when the study started. By 30 September 2024, at the end of this study, only 59 lung cancer patients were alive to complete the EORTC quality of life questionnaires. The study flowchart of lung cancer patients over time is exemplified in Figure 1.

The patients’ median age was 63 ± 9 years. Most of the patients (84.7%) had mNSCLC and only five NSCLC patients, who had advanced disease, were treated for consolidation therapy with Durvalumab. Only four SCLC patients were alive at the endpoint of the study. The demographic and clinical characteristics of the entire lung cancer patient population are thoroughly presented in Table 1.

Clinically relevant characteristics of the lung cancer patients at the baseline, such as their smoker status, body mass index, dyslipidemia and the presence of co-morbidities, such as Diabetes Mellitus, were registered, as detailed in Table 1. All NSCLC patients treated with Durvalumab for consolidation therapy had formerly received chemotherapy and radiotherapy. Thirty lung cancer patients (50.8%) had previously received curative-intent therapies such as surgery or radio-chemotherapy, with progression to advanced or metastatic disease before ICI initiation, as the first or second line of treatment. Treatment characteristics, such as the immunotherapy type and other treatment details, are shown in Table 2.

During the study, lung cancer patients were treated for infections or pain, using steroids and proton pump inhibitors (PPIs) for specific situations, such as palliative radiotherapy or emesis chemoprevention. Immune-related adverse events (irAEs) occurred in 19 lung cancer patients (32.2%). We also collected these data and presented them in Table 3.

Almost a third of the lung cancer cohort experienced irAEs during the study period, with most of them being endocrine-irAEs. We identified 13 cases of thyroiditis, 3 cases of hypophysitis, and 4 cases of primary adrenal insufficiency (PAI). All endocrine-irAEs were mild (Grade ≤ 2), except one case of moderate to severe (Grade ≥ 3) PAI. The average time on ICI therapy was 25.6 months. A summary of the irAEs of lung cancer patients treated with ICIs and evaluated for their quality of life using the EORTC QLQ is described in Table 4.

### 3.2. The Comparison of QoL Scores at the Start of ICI Treatment and at the Endpoint

Wilcoxon’s signed-rank test was applied to assess the differences between the QoL questionnaire scores at the start of ICI treatment and at our study’s endpoint on 30 September 2024. Most differences were statistically significant, excluding the insomnia, diarrhoea and financial difficulties scores for the QLQ-C30, and are thoroughly presented in Table 5.

**Figure 2:**
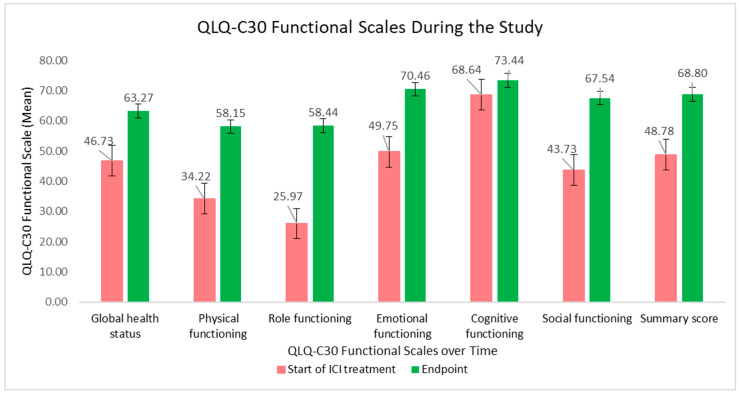
QLQ-C30 functional scales and summary score at the beginning of ICI treatment versus at the study endpoint.

The evolution of the QLQ-C30 functional scales is graphically presented in Figure 2. The global health status and physical, role, emotional and social functioning were significantly improved during ICI treatment, influencing the final summary score.

**Figure 3:**
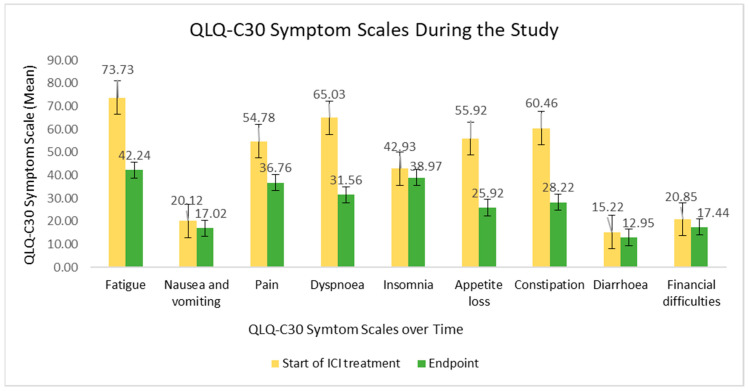
QLQ-C30 symptom scale at the beginning of ICI treatment versus at the study endpoint.

The QLQ-C30 symptom score evolution is clearly shown in Figure 3. Symptoms, such as fatigue, pain, dyspnoea, appetite loss and constipation were considerably decreased during ICI therapy, meaning an enhanced QoL. Overall, the least affected items were cognitive functioning, nausea and vomiting, insomnia and diarrhoea symptoms, besides financial difficulties.

When we compared the QLQ-LC13 scores, significant differences were found for all dyspnoea scores, coughing, pain in chest, pain in other parts and peripheral neuropathy, as specified in Table 6.

**Figure 4:**
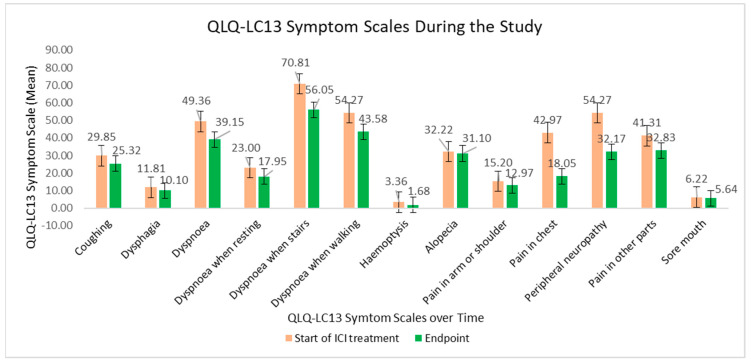
QLQ-LC13 symptom scales at the beginning of ICI treatment versus at the study endpoint.

The QLQ-LC-13 symptom scores during the study are visibly displayed in Figure 4. The decline in the scores over time meant a substantial recovery of quality of life. Lung-cancer-specific symptoms, such as dyspnoea and pain in chest, pain in arm and pain in shoulder, showed statistically significant declines.

### 3.3. The Influence of ICI-Related Predictors on the QoL Scores

We applied Mann–Whitney tests where the normality assumption was not met and independent samples Student’s T-tests when the data had a normal distribution. Only statistically significant results are shown in Table 6. We investigated whether any of the following predictors influenced any of the QLQ-C30 and QLQ-LC13 scores: endocrine-irAEs; any type of irAEs; steroids, PPIs or opioids being administered during ICI therapy; infections being treated during ICI therapy; the response to ICIs; and surgery before ICI therapy. For the latter, there were no significant differences in the questionnaire scores.

Suffering from endocrine-irAEs was associated with a worse cognitive function and a worse result in the coughing score. When considering every irAE, the pain score was better in patients with adverse effects, while the coughing score remained worse.

Managing specific clinical conditions by using steroids, PPIs and opioids during ICI therapy was associated with worse scores for nausea and vomiting. Considering only steroids and PPI treatment, both were associated with appetite loss, while steroids alone were associated with a greater score for dyspnoea when resting.

Patients who had infections treated while also being administered ICIs scored better on the QLQ-LC13 dyspnoea scale, specifically on the dyspnoea when stairs scale.

The response to ICIs was associated with worse scores on the pain in other parts and coughing scales. First-line ICI therapy resulted in worse scores on the following scales: physical functioning, fatigue, nausea and vomiting, pain and the summary score. The influence of ICI-related predictors on the QoL scores is illustrated in Table 7.

**Figure 5:**
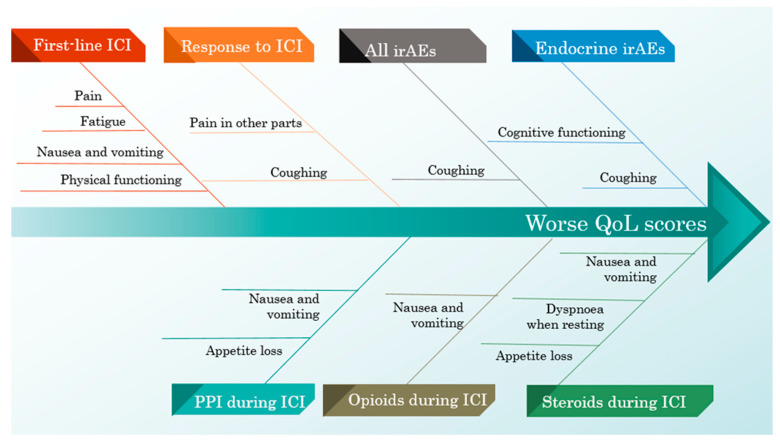
A cause–effect diagram illustrating the negative influence of ICI predictors on the QoL scores.

**Figure 6:**
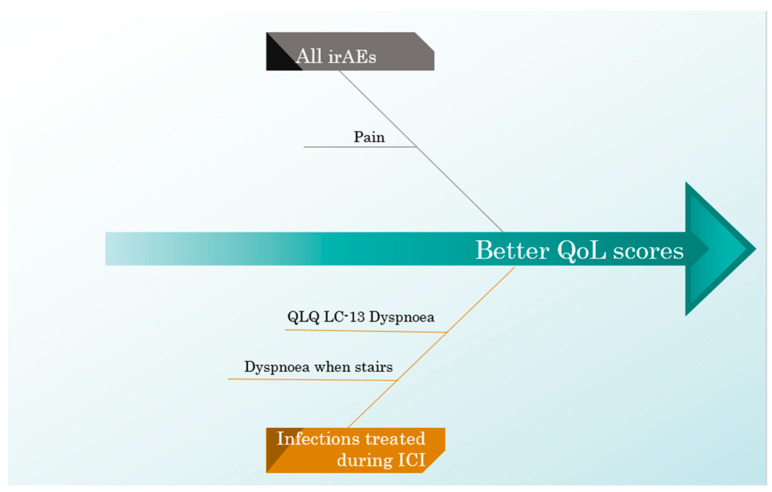
A cause–effect diagram illustrating the positive influence of ICI predictors on the QoL scores.

The negative and positive influences of all ICI predictors on the QoL scores are exemplified in Figure 5 and Figure 6.

### 3.4. Influence of Age and ICI Treatment Duration on QoL Scores

Taking into consideration that our study design employed mostly older patients who had been followed over different periods, we built a multiple linear regression model including the following independent variables: the number of weeks on ICI treatment, age and the baseline QoL score. This method was employed in order to see whether age and the treatment duration were significant predictors of the endpoint QoL scores when adjusting for the baseline scores.

Although every model was statistically significant as a whole, the age or weeks on ICIs were significant only for the scales presented in Table 8. An increasing age seemed to significantly lower the nausea and vomiting score, while a greater number of weeks on ICIs was associated with less pain in arm or shoulder.

## 4. Discussion

Despite considerable international research efforts towards implementing personalized screening programs and smoking cessation interventions [32], lung cancer persists as the leading cause of cancer death worldwide [1] and also in Romania [3], defying the medical community. Additionally, lung cancer will remain a major public health issue as the nation-wide screening program is still in the project phase [33] and smoking habits are increasing in young adults [34]. Nevertheless, a slight decrease in the lung cancer mortality rate from 27.4% in 2020 to 26.6% in 2022 [2,3] has shown the crucial progress in lung cancer therapy.

The innovative breakthrough of immunotherapy in lung cancer started over a decade ago with the anti-PD-1 antibody Nivolumab in the CheckMate 017 and 057 randomized clinical trials (RCTs) [13,35]. Nivolumab significantly improved the overall survival (OS) for metastatic non-small-cell lung cancer (mNSCLC) patients in the second line of palliative treatment versus chemotherapy (ChT). Since Nivolumab changed the paradigm and standard of care for mNSCLC patients, several new immune checkpoint inhibitors (ICIs) have revolutionized the treatment of non-oncogene-addicted lung cancer. Nowadays, in first-line PD-L1-positive [36,37,38] or PD-L1-negative mNSCLC patients [8,9,39,40,41,42] or those with unresectable locally advanced NSCLC after definitive chemoradiation [5], ICIs represent cutting-edge technology, being the first choice of treatment highly endorsed by international guidelines [4,6,7]. The newest published data from the PEARLS/KEYNOTE-091 study evidenced impressively augmented disease-free survival compared with a placebo in the adjuvant setting for completely resected stage IB-IIIA NSCLC patients [43]. The shift is moving even further from the metastatic stage to the earlier stages, as the CheckMate 816 trial reported a significantly improved pathological complete response with Nivolumab plus platinum-based ChT used for three cycles as neoadjuvant therapy in stage IB-IIIA NSCLC patients compared with ChT alone (24.0% versus 2.2%; odds ratio: 13.94; 99% CI: 3.49–55.75; *p* < 0.001) [44]. Immunotherapy also revealed huge efficacy and success in the most aggressive type of lung cancer, small-cell lung cancer (SCLC), after decades of discouraging results with standard chemotherapy [15,16], being approved by international regulatory authorities [14,45]. To summarize, more and more lung cancer patients, in different stages of the disease, will be treated and live longer using ICIs, and the balance between efficacy and safety will demand fairly accurate attention to prevent and treat toxicity issues. In Romania, immunotherapy in mNSCLC patients has been used since 1 November 2017, when Nivolumab was approved for second-line palliative treatment. Gradually, over the years, more ICIs have been approved for first-line mNSCLC or as consolidation therapy in unresectable locally advanced NSCLC after chemoradiation and for SCLC patients by The National Regulatory Authorities [46], but not all European Society of Medical Oncology (ESMO) guideline recommendations [6,14] or National Comprehensive Cancer Network (NCCN) guideline-approved indications are reimbursed [7,45]. Our cohort study evaluating quality of life comprised only 59 lung cancer patients, mostly first-line, ICI-treated mNSCLC patients. In light of the reimbursement constraints and late approvals in Romania and consequently the lack of clinical experience with new ICIs, this reality might explain the small, heterogeneous lung cancer cohort in our study. Additionally, being a tertiary level-hospital in Romania and not a high-volume lung cancer institute, of the 239 lung cancer patients treated in almost seven years, we succeeded in including and investigating in a prospective non-interventional study only 59 patients. These real-life data, although limited, might provide and point out the limitations and challenges that the medical community faces in real-world practice, in comparison with multicentered, international RCTs.

ICIs exert their antitumor effects by reducing the inhibition that cancer cells apply to immune checkpoint pathways to evade the host immune system. Enhancing the immune system’s ability to fight against tumor cells, ICIs also bring the expected risk of adverse consequences of the loss of immune tolerance, so-called immune-related adverse events (irAEs). Autoimmune toxicity may affect any organ, tissue or system; may be rare and serious, such as myocarditis, or frequent and minor, such as skin or endocrine toxicities; and, moreover, may appear early or late during immunotherapy. Our prospective exploratory study reported few other irAEs, three cases of autoimmune hepatitis, one case of colitis and one case of anemia, aligned with the rare incidence of non-endocrine-irAEs reported in RCTs [9,10]. The grading and management of irAEs are standardized by international guidelines [29,30,31].

Immune-mediated endocrinopathies are the most relevant and intriguing for long-survival lung cancer patients treated with ICIs since they are the most frequently reported irAEs, in roughly 10% of patients [47]. Furthermore, endocrine-irAEs generally tend to become irreversible and need life-long hormonal therapy [17]. Hypophysitis, thyroid disorder, primary adrenal insufficiency (PAI) and type 1 Diabetes Mellitus (DM) are the most regularly occurring autoimmune endocrinopathies during treatment with ICIs [48]. Worsening type 2 DM or the new onset of insulin-dependent DM is a rare event during ICI therapy; however, caution must be paid to properly diagnose and treat a life-long irAE. Immune-mediated hypophysitis typically affects the anterior pituitary gland, involving several hormonal axes and implying multiple deficiencies, such as thyroid, adrenal or gonadotropic insufficiencies. Even if thyroid function may improve in the long term, corticotrope cell function recovery is rare, so the quality of life during or after ICI therapy represents a vital concern for patients with secondary adrenal insufficiency [47,49]. Primary adrenal insufficiency (PAI), like secondary adrenal insufficiency, may present with non-specific or cancer-specific symptoms (nausea, abdominal pain, vomiting, anorexia, weight loss, fatigue and hypotension), as well as an adrenal crisis, a life-threating condition involving shock, confusion and electrolyte abnormalities. Morning serum cortisol and adrenocorticotropin (ACTH) levels, serum electrolyte levels and, for inconclusive cases, a cosyntropin test are diagnostic for PAI [49,50]. Last but not least, thyroid dysfunction is one of the most frequent endocrine-irAEs in NSCLC patients treated with ICIs, and hypothyroidism needs life-long hormonal substitution [51,52]. The major scientific interest in endocrine-irAEs and especially thyroiditis is augmented by substantially increasing evidence of a predictive correlation between autoimmune endocrinopathies and ICI efficacy [19,20,21,53,54,55,56,57,58,59]. Nevertheless, conflicting results on low-strength links have been published [60,61]. In our prospective study, 33.8% of lung cancer patients encountered endocrine-irAEs with a positive impact on their quality of life, meaning a longer and better life with endocrine-irAEs.

The quality of life of metastatic lung cancer patients stands as a crucial value in overall treatment management, influencing both therapy decisions and efficacy assessments. Even though in metastatic disease there are only two major endpoints, overall survival and quality of life, not all ICI RCTs have evaluated quality of life besides toxicity-related secondary endpoints [37,62], or QoL has only been investigated in an exploratory way [63,64], QoL data are pending [38,39] or they have shown no improvements in QoL estimations [11,65,66]. Nonetheless, ICIs are already approved by regulatory authorities and used in clinical practice, mostly based on their progression-free survival benefits and toxicity profiles [4,6,14,46]. Traditionally, the lung-cancer-specific quality of life in clinical trials has been investigated using the EORTC QLQ-LC13 as the standard instrument for evaluating and monitoring symptoms and the global health status during randomized trials [25]. The KEYNOTE-024 study reported in 2017 health-related quality of life results for treatment with pembrolizumab versus chemotherapy in advanced PD-L1-positive mNSCLC patients using the EORTC QLQ-C30 and EORTC QLQ-LC-13 [26], proving a sustainable QoL status during immunotherapy. Nowadays, there is extensive evidence supporting the utility and positive function of patient-reported outcome measures (PROMs) across the continuum of cancer care [24]. The evaluation of quality of life during research is moving towards the use of PROMs as cancer patients receiving active cancer therapies are dynamically involved in their care and healthcare providers monitor symptom management in real time. On the other hand, the integration of PROMs into routine clinical practice and especially into long-term survivorship surveillance might face noteworthy barriers in the form of a lack of technological infrastructure and trained medical personnel or older patients’ lack of ability to complete PROMs. The Lung Cancer Europe (LuCE) Organization recently reported a descriptive research analysis of the social, psychological and medical impacts of lung cancer in patients across Europe [67]. Unfortunately, in Romania, the level of information and knowledge concerning disease and decision-making implications were impressively low, raising concerns about prognostic and treatment success and profoundly affecting emotional and social functioning [68].

In our prospective research, which included 59 lung cancer patients treated with ICIs and evaluated for their quality of life using the EORTC’s QLQ-C30 and QLQ-LC-13, we analyzed their global health status, functional scales and general and lung-cancer-specific symptom scores before receiving ICIs and at the endpoint of the study. Additionally, we investigated several predictors of the potential impact upon quality of life during ICI therapy.

The overall quality of life was statistically significantly improved during immunotherapy, especially regarding physical, role, emotional and social functioning. General symptoms, such as pain, fatigue, dyspnoea, appetite loss and constipation, were also impressively relieved. Furthermore, the value added to quality of life during ICI therapy was evidence of a declining trendline for lung-cancer-specific symptoms, such as coughing, dyspnoea (when resting, when stairs, when walking), hemoptysis, pain (in arm or shoulder, in chest, in other parts) and peripheral neuropathy. Generally, immunotherapy was associated with a better quality of life for lung cancer patients during the study. Although a small and heterogeneous cohort population was included, this research proved the validity and utility of assessing quality of life during treatment besides the routine evaluation of treatment-specific toxicity.

As has already been conveyed, co-medication during immunotherapy might have a negative impact on ICI efficacy. The long-term usage of proton pump inhibitors [69,70,71] or steroids during ICI therapy [72] or opioids for pain management [73] was correlated with poorer clinical outcomes. The dismal influence of co-medications on the quality of life of lung cancer patients treated with ICIs was confirmed in our prospective study. Although treated infections during ICI treatment were proven to negatively influence the efficacy of the response [74], in our quality of life research, a positive effect on dyspnoea as a lung-cancer-specific symptom was shown.

Endocrine-irAEs affected the QoL of the lung cancer cohort in both negative and positive ways, improving pain and worsening cognitive functioning and coughing. As toxicities themselves, this duality is relatively reasonable and physiologically understandable. Nevertheless, due to the impressive disease control, meaning enhanced PFS, OS and value-added symptom control, achieved by immunotherapy, lung cancer patients experiencing endocrine-irAEs also showed a high quality of life. Their overall quality of life was boosted by ICI treatment during our study. Bearing in mind the value of the predictive biomarkers of endocrine-irAEs for ICI responses in cancer patients [19,20,21,53,54,55,56,57,58,59], the rising number of cancer sites treated with immunotherapy, new indications and the incidence of cancer worldwide, the evaluation of the quality of life in these challenging populations developing endocrine-irAEs unveils a new scientific research perspective and future directions for investigation. These cancer patient populations live longer and can even be cured with ICIs in early cancer stages but survive with endocrine-irAEs, needing life-long hormonal substitution and a good quality of life. The literature revealed scarce data about the QOL of lung cancer patients treated with immunotherapy and concomitantly experiencing endocrine-irAEs, and this small-cohort prospective study established a first step forward in this direction. 

Currently, there is an apprehensive focus on elaborating special questionnaires for assessing QoL in patients undergoing immunotherapy. The EORTC is developing a questionnaire aiming to identify health-related quality of life issues and create a methodology for measuring these aspects in cancer patients treated with immunotherapy, the QLQ-ICI, a useful future tool for upcoming exploration [27]. Our study is aligned with other prospective non-interventional research investigating the questionnaire-based detection of irAEs in cancer patients treated with immunotherapy [75,76,77].

Our research has several limitations to be listed. First of all, our prospective study comprised a small heterogenous cohort of lung cancer patients. Secondly, we used versions of the EORTC QLQ-C30 and QLQ-LC-13 validated in and translated into the Romanian language, as PROMs are not yet standardized in Romania. Thirdly, the statistically significant predictors and outcomes need more exploration in a larger homogenous cohort. Finally, a personalized immunotherapy questionnaire would assess precise items more relevant to clinical practice. Nevertheless, our small prospective cohort study fulfilled its purpose of highlighting the emerging need to explore quality of life, especially in cancer patients with endocrine-irAEs, as they live longer with life-long hormonal treatment.

## 5. Conclusions and Future Directions

An appropriate assessment of the quality of life of lung cancer patients is needed to optimize the management of toxicities, especially immune-related adverse events. Our research showed clear evidence and statistically significant improvements in the quality of life of lung cancer patients treated with ICIs, even those with endocrine-irAEs. As extensive new indications of immunotherapy will affect early-stage lung cancer patients, with curative intent, we draw attention to this particular population living longer with autoimmune endocrinopathies in need of life-long hormonal therapy.

## Figures and Tables

**Table 1:** Demographic and clinical characteristics of lung cancer patients treated with ICIs and evaluated using EORTC QLQs.

Characteristic	*n* = 59
Age	
Mean—years	63 ± 9
≥65 years, n (%)	30 (50.8)
<65 years, n (%)	29 (49.1)
Male sex—no. (%)	35 (59.3)
Smoker—no. (%)	
Yes	31 (52.5)
No	28 (47.5)
Body mass index (kg/m^2^)—no. (%)	
<18.5	2 (3.4)
≥18.5 < 24.9	21 (35.5)
≥24.9	36 (61)
Dyslipidemia	
Yes	31 (52.5)
No	28 (47.4)
Diabetes Mellitus	
Yes	7 (11.8)
No	52 (88.1)
ECOG performance status 0–2	59
Histologic features—no. (%)	
Non-small-cell lung cancer	55 (93.2)
Adenocarcinoma	36 (61.0)
Squamous cell carcinoma	19 (32.2)
Small-cell lung cancer	4 (6.7)
Lung cancer TNM stage	
Non-small-cell lung cancer	55 (93.2)
Stage III	5 (8.5)
Stage IV	50 (84.7)
Small-cell lung cancer	4 (6.7)
Stage III	1 (1.7)
Stage IV	3 (5.0)
PD-L1 Tumor Proportion Score in NSCLC patients—no. (%)	
<1%	15 (25.4)
1–49%	14 (23.7)
≥50%	24 (40.6)
Not assessed	2 (3.4)

**Table 2:** Treatment characteristics of lung cancer patients treated with ICIs and evaluated by EORTC QLQs.

Immunotherapy Type and Treatment Details	*n* = 59
ICIs used and line of treatment—no. (%)	
Advanced NSCLC patients	5 (8.5)
Durvalumab—consolidation therapy	5 (8.5)
Metastatic NSCLC patients	50 (84.7)
First-line	45 (76.2)
Pembrolizumab/ChT	24 (40.6)
Pembrolizumab monotherapy	14 (23.7)
Nivolumab/ipilimumab + ChT	7 (11.9)
Second-line	5 (8.5)
Nivolumab	3 (5)
Atezolizumab	1 (1.7)
Pembrolizumab	1 (1.7)
SCLC patients	4 (6.7)
Atezolizumab	2 (3.4)
Durvalumab	2 (3.4)
Average time on ICIs (range)—months	17.7 ± 18.2
Average time on ICIs (range)—weeks	76.02 ± 77.8
Average number of ICI cycles (range)	26.4 ± 28.5
ICI treatment response ^1^—no. (%)	
Complete response	1 (1.7)
Partial response *	44 (74.5)
Progressive disease	4 (6.7)
Not assessed	10 (16.9)
Treatment before ICIs ^2^—no. (%)	30 (50.8)
Surgery	18 (30.5)
RT-ChT/ChT	12 (20.3)
Palliative radiotherapy ^3^—no. (%)	19 (32.2)
Average time on total treatment ^4^ (range)—months	26.9 ± 22
Average time from Dg to the end of the study ^5^ (range)—months	30.1 ± 2.9

^1^ Patients were evaluated using RECIST v1.1. * Partial response was assessed as a partial response, stable disease, a clinical benefit or a combination of these. ^2^ Chemotherapy at the physician’s choice, using docetaxel, gemcitabine, navelbine, paclitaxel or pemetrexed. ^3^ Patients were treated with RT for tumors and mediastinal lymph nodes or brain or bone metastases. ^4^ Total treatment was composed of all curative and palliative treatments during the study. ^5^ The end of the study was 30 September 2024. RT, radiotherapy; ChT, chemotherapy; Dg, diagnosis.

**Table 3:** Clinical features of lung cancer patients treated with ICIs and evaluated using EORTC QLQs.

Characteristic	*n* = 59
Treated infections during ICI therapy	
Yes	13 (22)
No	46 (78)
Steroids during ICI therapy	
Yes	27 (45.7)
Brain metastasis	8 (13.5)
Radiotherapy for brain or bone metastasis	12 (20.3)
Chemoprevention of emesis	24 (40.6)
No	32 (54.2)
PPIs during ICI therapy	
Yes	31 (52.5)
No	28 (47.4)
Opioids during ICI therapy	
Yes	11 (18.6)
No	48 (81.3)
Immune-related adverse events ^1^	19 (32.2)
Endocrine irAES	17 (28.8)
Other irAES	2 (3.4)

^1^ Three patients developed both endocrine and non-endocrine irAEs.

**Table 4:** Summary of irAEs of lung cancer patients treated with ICIs and evaluated using EORTC QLQs.

Characteristic	*n* = 19			
Average time to irAEs (range)—months	7 ± 10.9			
Average time on ICIs (range)—months	25.6 ± 16.9			
Average time on ICIs (range)—weeks	109.8 ± 72.4			
Average number of ICI cycles (range)	39 ± 28.1			
Average time on total treatment ^a^ (range)—months	38 ± 20.4			
Average time from Dg to the end of the study ^b^ (range)—months	41.55 ± 20.8			
**Endocrine-irAES ^1,2^**	**20 (33.8)**	**irAEs of Grade ≤ 2**	**irAEs of Grade ≥ 3**	**Treatment**
Thyroiditis	13 (22)	13 (22)	0	Levothyroxine as Needed
Hypothyroidism ^3^	10 (17)	10 (17)	0	Levothyroxine
Hyperthyroidism	3 (5)	3 (5)	0	None
Hypophysitis ^4^	3 (5)	3 (5)	0	Levothyroxine/Prednisone
Primary Adrenal Insufficiency	4 (6.7)	3 (5)	1 (1.7)	Prednisone
**Other irAES**	**5 (8.5)**	2 (3.4)	3 (5)	
Hepatitis	3 (5)	1 (1.7)	2 (3.4)	Methylprednisolone
Colitis	1 (1.7)	1 (1.7)	0	Loperamide
Anemia	1 (1.7)	0	1 (1.7)	Methylprednisolone

^1^ Three patients developed both endocrine and non-endocrine irAEs. ^2^ Three patients experienced more than one affected gland. ^3^ Patients who had subclinical hypothyroidism did not need therapy. ^4^ One patient experienced hypophysitis involving thyroid, adrenal and gonadotropic deficiencies and needed hormonal replacement therapy for all three glands. ^a^ Total treatment was composed of all curative and palliative treatments during the study. ^b^ The end of the study was 30 September 2024.

**Table 5:** QLQ-C30 scores at the start of immunotherapy versus at the study endpoint.

QoL Score	Start of ICI Treatment		Endpoint		Wilcoxon	
	Mean	Std. Deviation	Mean	Std. Deviation	W	Sig. (p)
Global health status	46.73	14.83	63.27	20.55	5	<0.001
Physical functioning	34.22	16.16	58.15	19.8	0.00	<0.001
Role functioning	25.97	22.61	58.44	29.82	0.00	<0.001
Emotional functioning	49.75	20.64	70.46	22.63	0.00	<0.001
Cognitive functioning	68.64	27.72	73.44	27.88	0.00	0.020
Social functioning	43.73	24.35	67.54	24.84	0.00	<0.001
Fatigue	73.73	22.45	42.24	25.52	1770	<0.001
Nausea and vomiting	20.12	23.74	17.02	23.5	21	0.034
Pain	54.78	25.8	36.76	26.81	1400	<0.001
Dyspnoea	65.03	24.45	31.56	27.36	1653	<0.001
Insomnia	42.93	35.13	38.97	36.25	15	0.057
Appetite loss	55.91	23.73	25.91	29.11	1326	<0.001
Constipation	60.46	29.48	28.22	29.64	1431	<0.001
Diarrhoea	15.22	25.03	12.95	21.46	10	0.095
Financial difficulties	20.85	27.63	17.44	24.25	15	0.057
Summary score	48.78	14.9	68.8	16.44	0.00	<0.001

**Table 6:** QLQ-LC13 scores at the start of immunotherapy versus at the endpoint.

QoL Score	Start of ICI Treatment		Endpoint		Wilcoxon	
	Mean	Std. Deviation	Mean	Std. Deviation	W	Sig. (p)
Coughing	29.85	26.82	25.32	23.48	28	0.020
Dysphagia	11.81	22.09	10.1	19.75	3	0.371
Dyspnoea	49.36	18.48	39.15	22.78	325	<0.001
Dyspnoea when resting	23	21.62	17.95	19.81	45	0.005
Dyspnoea when stairs	70.81	18.68	56.05	28.11	210	<0.001
Dyspnoea when walking	54.27	23.29	43.58	29.36	136	<0.001
Hemoptysis	3.36	10.06	1.68	7.31	6	0.149
Alopecia	32.22	33.41	31.1	31.59	3	0.346
Pain in arm or shoulder	15.2	24.23	12.97	24	10	0.072
Pain in chest	42.97	32.9	18.05	25.06	666	<0.001
Peripheral neuropathy	54.27	26.36	32.17	30.39	561	<0.001
Pain in other parts	41.3	41.74	32.83	33.73	105	<0.001
Sore mouth	6.22	20.07	5.64	18.74	1	1.000

**Table 7:** The influence of ICI-related predictors on the QoL scores.

QoL Score	Endocrine-irAEs (Number)		Mann–Whitney *p*-Value
	Yes (17)	No (42)	
Cognitive functioning	63.65 (25.92)	77.40 (27.96)	0.043
Coughing	41.12 (22.35)	18.93 (20.96)	0.001
	**All irAEs (Number)**		
	**Yes (19)**	**No (40)**	
Pain	26.42 (23.81)	41.67 (27.03)	0.036
Coughing	36.79 (24.74)	19.87 (21.04)	0.013
	**Steroids During ICI Therapy**		
	**Yes (27)**	**No (32)**	
Nausea and vomiting	24.78 (24.68)	10.47 (20.63)	0.007
Appetite loss	33.26 (27.83)	19.72 (29.15)	0.032
Dyspnoea when resting	24.56 (21.87)	12.37 (16.23)	0.028
	**Opioids During ICI Therapy**		
	**Yes (11)**	**No (48)**	
Nausea and vomiting	34.91 (28.47)	12.92 (20.42)	0.010
	**PPIs During ICI Therapy**		
	**Yes (31)**	**No (28)**	
Nausea and vomiting	22.13 (24.17)	11.36 (21.77)	0.035
Appetite loss	33.26 (29.89)	17.79 (26.41)	0.027
	**Infections Treated During ICI Therapy**		
	**Yes (13)**	**No (46)**	
QLQ LC-13 dyspnoea	28.92 (27.04)	42.04 (20.86)	0.045
Dyspnoea on stairs	38.46 (33.02)	61.02 (24.77)	0.015
	**Response to ICIs**		
	**Yes (45)**	**No (14)**	
Pain in other parts	38.6 (34.17)	14.29 (25.27)	0.019
Coughing	28.8 (24.28)	14.14 (16.95)	0.049
	**First-Line ICIs**		
	**Yes (48)**	**No (11)**	
Physical functioning	54.94 (19.45)	72.18 (15.11)	0.008 *
Fatigue	45.67 (25)	27.27 (23.16)	0.032
Nausea and vomiting	19.85 (24.98)	4.64 (7.94)	0.050
Pain	39.96 (26.4)	22.82 (25.09)	0.048
Summary score	66.76 (15.97)	77.71 (16.21)	0.038 *

* Normal distribution assumption met; *p*-value calculated with independent samples Student’s T-test.

**Table 8:** Multiple linear regression model.

QoL Score	Age		Weeks of ICI Treatment		Score at the Start of ICI Treatment			
	β	Sig. (p)	β	Sig. (p)	β	Sig. (p)	Adj. R^2^	*p*-Value
Nausea and vomiting	−0.36	0.012	0.01	0.711	0.92	<0.001	0.84	<0.001
Pain in arm or shoulder	−0.01	0.926	−0.03	0.037	0.94	<0.001	0.89	<0.001

## Data Availability

The data are available on request due to ethical restrictions. The data presented in this study are available on request from the corresponding author. The data are not publicly available due to the policy of the Coltea Clinical Hospital requiring the approval of the Ethics Committee for each new research study.

## Abbreviations

The following abbreviations are used in this manuscript:

QOL	Quality of life
RCTs	Randomized clinical trials
EORTC	European Organisation for Research and Treatment of Cancer
EORTC QLQ-C30	EORTC Core Quality of Life Questionnaire Core 30 items
EORTC QLQ-L13	EORTC Quality of Life Questionnaire-Lung Cancer 13 items
CTLA-4	Cytotoxic T-lymphocyte-associated antigen 4
PD-1/PD-L1	Programmed cell death 1/ligand pathways
NSCLC	Non-small-cell lung cancer
SCLC	Small-cell lung cancer
ICI	Immune checkpoint inhibitor
irAE	Immune-related adverse event
ACTH	Adrenocorticotropic hormone
TFT	Thyroid functional test
PPI	Proton pump inhibitor
DM	Diabetes Mellitus
PAI	Primary adrenal insufficiency
